# Rituximab in treatment of anti-GBM antibody glomerulonephritis: A case report and literature review

**DOI:** 10.1097/MD.0000000000017801

**Published:** 2019-11-01

**Authors:** Mayu Uematsu-Uchida, Takehiro Ohira, Shigeki Tomita, Hiroshi Satonaka, Akihiro Tojo, Toshihiko Ishimitsu

**Affiliations:** a Department of Nephrology & Hypertension, Dokkyo Medical University, Mibu, Tochigi; b Department of Pathology, Juntendo University Urayasu Hospital, Urayasu, Chiba, Japan.

**Keywords:** anti-glomerular basement membrane disease, CD20, CD4, collagen IVα3, rituximab

## Abstract

**Rationale:**

Anti-glomerular basement membrane (GBM) disease is a T cell-mediated disease that has a poor prognosis with conventional therapy. We tested rituximab as a primary therapy to reduce anti-GBM antibody produced by B cells.

**Patient concerns:**

A 53-year old woman with complaints of a fever, headache and abdominal discomfort showed renal failure with elevated anti-GBM antibody, and renal biopsy revealed crescentic necrotizing glomerulonephritis with linear immunoglobulin G (IgG) 1 deposition along GBM.

**Diagnoses:**

The patient's plasma contained autoantibodies against Goodpasture antigen, which is the NC domain of collagen IVα3, and CD4-positive helper T cells were found surrounding crescent glomeruli with the coexistence CD20-positive B cells.

**Interventions:**

Rituximab with steroid and plasma exchange.

**Outcomes:**

The levels of autoantibody for Goodpasture antigen were reduced, and the patient was able to temporarily withdraw from hemodialysis.

**Lessons:**

B cell depletion with rituximab is effective as an initial therapy for anti-GBM disease.

## 1 Introduction

Anti-glomerular basement membrane (GBM) disease is an autoimmune disease, and the primary target of circulating and in situ bound antibody is the non-collagenous (NC) 1 domain of the α3 chain of type IV collagen.^[1,2]^ This antigen is known as the Goodpasture antigen, and the anti-GBM autoantibodies not only damage the glomerular GBM, resulting in rapidly progressive glomerulonephritis with crescent formation via the ruptured GBM, but also disrupt the alveolar basement membrane, resulting in pulmonary hemorrhaging. Patients with Goodpasture syndrome have autoantibodies against Goodpasture antigens, including α3 (IV) NC1 domain, α1 (IV) NC1 and α4 (IV) NC1 at frequencies of 80%, 15% and 4%, respectively.^[3]^

The native form of Goodpasture antigen does not bind to B cell receptors, but CD4+ helper T cells recognize Goodpasture antigen in the linear peptide form bound to human leucocyte antigen (HLA) class II molecule on surface antigen-presenting cells.^[4,5]^ Anti-GBM disease is therefore considered a type of T cell disorder disease in the certain HLA such as HLA-DR15 with DNA type HLA-DRB1∗1501.^[4–6]^

It was recently reported that B cell depletion by rituximab was effective for anti-GBM disease.^[7,8]^ We herein report a case of anti-GBM disease with hemodialysis, in which B cell depletion by rituximab was effective in allowing the patient to withdraw from hemodialysis for a while. We also discuss and review the underlying mechanism and regimen of treatment for anti-GBM disease.

## 2 Case study

A 53-year old woman visited our hospital with complaints of a fever, headache and abdominal discomfort. She was healthy before and had nothing particular in her family history. A physical examination showed no abnormalities except for a slight fever of 37.1°C. Urinalysis revealed proteinuria 1.14 g/gCr with elevated numbers of red blood cells per high-power field (HPF), white blood cells 10 to 19/HPF and white blood cell casts. The renal function was severely deteriorated with serum creatinine levels of 8.86 mg/dL, an estimated glomerular filtration rate of 4.2 mL/min/1.73 m^2^ and urea nitrogen level of 75 mg/dL. She had anemia, with a hemoglobin level of 8.7 g/dl, white blood cell count of 8500/μL, and platelet count of 44.6 × 10^4^/μL. Serological test showed increased C-reactive protein levels at 25.59 mg/dL with normal immunoglobulin, complements and antinuclear antibodies. Myeloperoxidase (MPO)-anti-neutrophil cytoplasmic antibody (ANCA) and proteinase 3 (PR3)-ANCA were negative, but anti-GBM antibodies were extremely elevated at 1170 U/mL. Chest X-ray and computed tomography did not show pulmonary hemorrhaging or interstitial fibrosis. The kidney size was normal (9.7 × 4.1 cm in the right kidney and 9.5 × 4.7 cm in the left kidney).

**Figure 1:**
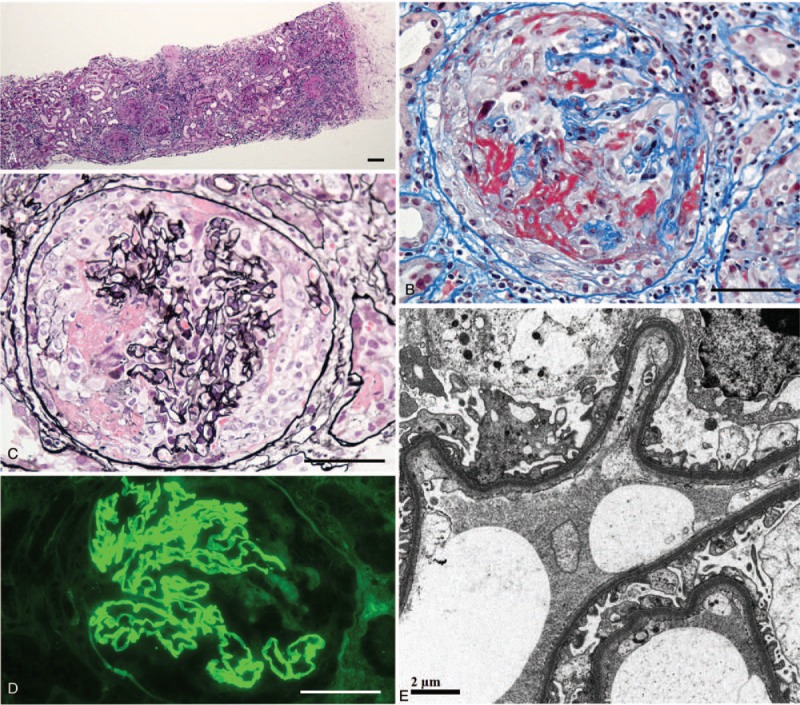
Renal biopsy samples with PAS staining (A), Azan staining (B), PAM staining (C), immunofluorescence for IgG (D) and electron microscopy (E). The bars indicate 100 μm (A) and 50 μm (B–D).

A renal biopsy and sample analysis were performed with the approval of the research ethics committee of Dokkyo Medical University (R-2–1), and revealed diffuse necrotizing crescentic glomerulonephritis (13/24 = 54%) with interstitial lymphocyte infiltration on Periodic acid Schiff (PAS) staining (Fig. 1A). Periodic acid methenamine silver (PAM) and Azan staining showed cellular crescents with GBM ruptures and fibrin deposition (Fig. 1B, C). Immunofluorescence showed linear IgG staining along the GBM (Fig. 1D), and electron microscopy demonstrated linear electron-dense deposition in the GBM (Fig. 1e). We diagnosed her with renal restrictive Goodpasture syndrome, that is, anti-GBM antibody glomerulonephritis.

**Figure 2:**
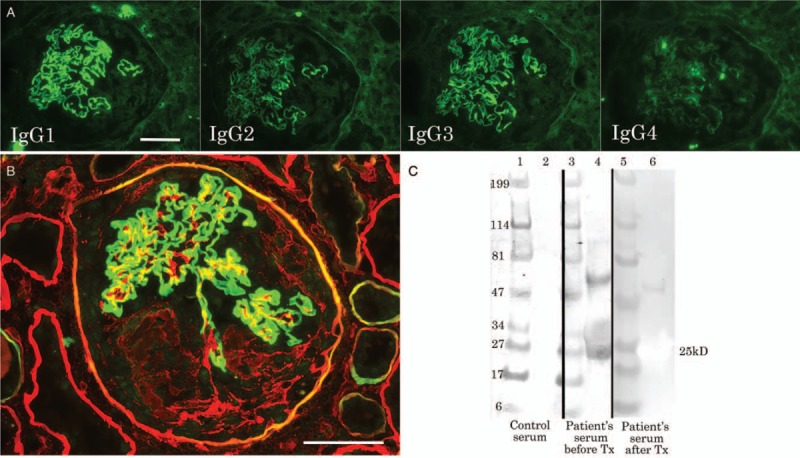
Immunofluorescence for IgG subclass (A), double staining for type IV collagen α2 (red) and α5 (green) (B) and immunoblotting for NC1 domain protein of type IV collagen α3 (lane 2, 4, 6) and molecular weight markers (lane 1, 3, 5) with the patient's serum before (3, 4) and after (5, 6) treatment as well as control serum (1, 2) (C). The bars indicate 50 μm.

Immunofluorescence of the IgG subclass revealed that IgG1 was most strongly stained, followed by IgG3, along the GBM with a linear pattern, whereas IgG2 and IgG4 were faintly stained along the GBM (Fig. 2A), suggesting poor prognosis. Double immunostaining with a mixture of Texas Red-labeled anti-human collagen IV α2 antibody and fluorescein isothiocyanate (FITC)-labeled anti-human collagen IV α5 antibody (Shigei Medical Research Institute, Okayama, Japan) revealed that Type IV collagen α5 was linearly stained along the GBM, Bowman's capsule and distal tubular basement membrane, whereas type IV collagen α2 was stained in the mesangium, fibers in the crescent, Bowman's capsule and tubular basement membrane (Fig. 2B). The localization and staining of Type IV collagen α5 was normal, suggesting not a target of disease like Alport syndrome.

To detect autoantibody for collagen type IV α3 (COL4A3) Goodpasture antigen in patient's serum, recombinant protein epitope signature Tag (PrEST) for human COL4A3 (Atlas antibodies, Stockholm, Sweden) was electrophoresed on a 4% to 20% polyacrylamide gel, and blotted on the polyvinylidene fluoride (PVDF) membrane. The membrane with 25-kD PrEST for human COL4A3 was incubated with patient serum before and after treatment, followed by incubation with HRP-labeled secondary antibody for anti-human IgG antibody (1:2000 dilution). The serum with lupus nephritis served as a control. Goodpasture antigen, NC domain of type IV collagen α3, was recognized in the patient's serum before treatment but not after treatment or serum from lupus nephritis (Fig. 2C).

**Figure 3:**
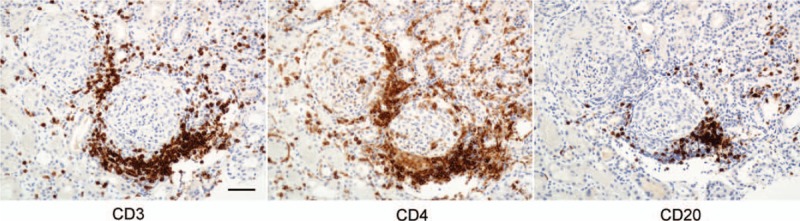
Immunostaining for CD3, CD4, and CD20. The bar indicates 50 μm.

Among the interstitial-infiltrating cells, CD4-positive helper T cells were predominantly located around the crescent glomeruli, and these helper T cells were colocalized with CD20-positive B cells (Fig. 3), indicating that both T cells and B cells play a role in anti-GBM disease.

**Figure 4:**
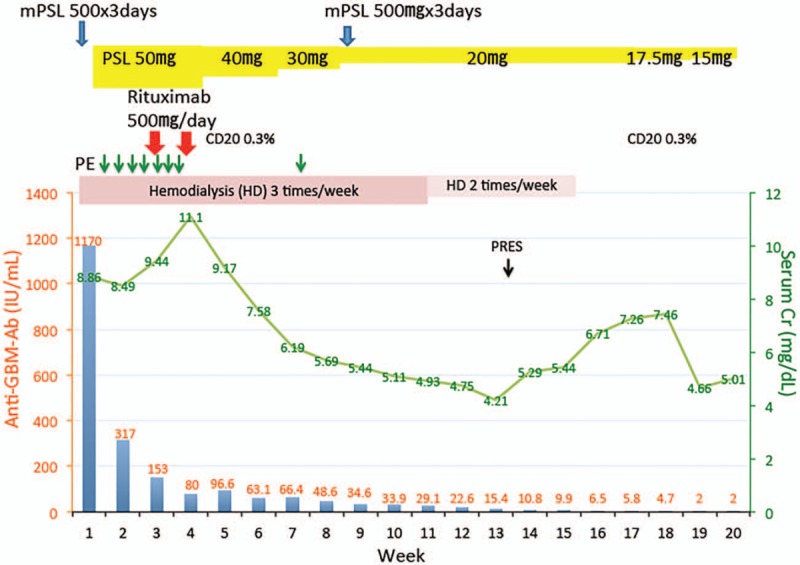
Clinical course of the renal function, anti-GBM antibody titer and number of CD20 cells during treatment with rituximab, plasma exchange and corticosteroid administration. PRES = posterior reversible encephalopathy syndrome, PE = plasma exchange, mPSL = methylprednisolone pulse therapy.

After the renal biopsy, she was immediately treated with intravenous injection of methylprednisolone 500 mg for 3 days followed by oral administration of prednisolone 50 mg/day (Fig. 4) for 4 weeks. Plasma exchange was performed 3 times a week for 3 weeks, and rituximab 375 mg/m^2^ (500 mg) was administered 2 and 3 weeks after admission. With this treatment, the anti-GBM antibody level decreased to 317 U/mL at 2 weeks and 153 U/mL at 3 weeks. However, the serum creatinine level increased to 11.10 mg/dL, so hemodialysis was maintained without plasma exchange. At 12 weeks, the titer of anti-GBM antibody reached 22.6 U/mL, and the serum creatinine level remained steady at 4.75 mg/dL, so hemodialysis was reduced to twice a week. At 13 weeks, the serum creatinine decreased to 4.21 mg/dL; unfortunately, however, posterior reversible encephalopathy syndrome (PRES) developed even though the systolic blood pressure was about 140 to 160 mmHg, and the serum creatinine level increased again. Hemodialysis was withdrawn at 16 weeks, and she was discharged from the hospital without hemodialysis at 18 weeks (Fig. 4). At 23 weeks, she was placed on maintenance hemodialysis.

## 3 Discussion

In our patient, we detected autoantibody against recombinant NC1 domain protein of Col4A3 in the plasma, which was consistent with previous reports. The titer of anti-GBM antibody was extremely high in our patient, and the serum after treatment with an anti-GBM antibody titer <2 U/mL did not react with recombinant NC1 domain protein of Clo4A3. It has been reported that certain HLA class II types, including HLA-DR15 (DNA type HLA-DRB1∗1501), are associated with increased levels of autoantibodies against NC1 domain protein of Col4A3.^[4–6]^ The present patient had HLA class II type with DR13 (DRB1∗13:01/02/07), DR15 (DRB1∗15:01/02), DR52, DR51 and DQ6, which is consistent with those previous findings.

The merit of our case report is that even though our patient had end-stage renal failure, treatment with plasma exchange, methylprednisolone pulse therapy and the early start of rituximab achieved partial remission and allowed for the temporary withdrawal of hemodialysis. A number of clinical and histological poor prognostic factors of anti-GBM antibody glomerulonephritis have been reported, including creatinine levels exceeding 500 to 600 μmol/L (5.7–6.8 mg/dL) and the need for hemodialysis at presentation as well as crescent >50% of glomeruli on renal biopsy samples.^[9–11]^ Approximately 10% to 40% of anti-GBM antibody glomerulonephritis patients were positive for anti-neutrophil cytoplasmic antibodies (ANCA), which exist before anti-GBM antibodies become positive, suggesting that ANCA may be related to the onset of the disease,^[12]^ and the renal prognosis was poor in those patients.^[10]^ The present case had a number of poor prognostic factors, including a serum creatinine level of 8.86 mg/dL with hemodialysis and 54% crescents, although ANCA was negative, suggesting a 1-year renal survival of <8%.^[10]^ We therefore administered rituximab injection soon after methylprednisolone pulse therapy and seven sessions of plasma exchange therapy.

Rituximab reduced the pan B cell count to 0.3% of total lymphocytes, and this reduction in CD20 B cells was still present at18 weeks. Mature T cells (CD3) and helper T cells (CD4) were co-localized with pan B cells around the crescentic glomeruli in Figure 3, suggesting that B cells received signals to produce anti-GBM antibodies, and rituximab continued to keep anti-GBM antibodies low at 19 weeks. The results of previous reports of the treatment of anti-GBM disease with rituximab are summarized in Table 1. About half of the cases treated with rituximab saw a restoration of the serum creatinine level 2 to 4 mg/dL in chronic kidney disease stage 3 to stage 4,^[7,8,13–16]^ although they initially needed hemodialysis, as in our case. One patient received rituximab at a dose of 375 mg/m^2^ twice at the beginning of treatment with prednisolone, and 17-time intensive plasma exchange showed complete remission with normal creatinine level of 1.1 mg/dL.^[8]^ In our case, it took 19 weeks to reduce the titer of anti-GBM antibody to <2 U/mL, so some progression of fibrosis was allowed, and the renal function could not be normalized. To normalize the renal function, it may be necessary to render the anti-GBM antibody titer negative as quickly as possible through intensive plasma exchange and the early use of rituximab.

**Table 1:**
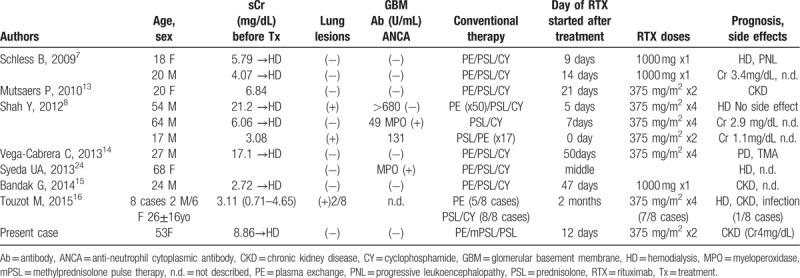
Case reports of anti-GBM antibody glomerulonephritis treated with rituximab.

The limitation of this report was that we could not restore the renal function to the normal level, and the patient ultimately had to be reintroduced to hemodialysis. It may be surmised that we should have repeated plasma exchange until the anti-GBM antibody titer becomes negative instead of stopping it after seven sessions in early treatments. PRES worsened the serum creatinine level. Even though PRES occurred 9 weeks after the final infusion of rituximab in our case, we should remember that rituximab has induced PRES soon after infusion in most cases,^[17–19]^ but in others, it occurred 10 days^[20]^ or 6 months after rituximab treatment.^[21]^

In conclusion, anti-GBM glomerulonephritis is believed to be a T cell disease, and increased populations of helper T cells stimulate B cells to produce antibodies against the NC domain of Col4A3. We report a case of anti-GBM glomerulonephritis treated with rituximab at the initial treatment in which the patient was able to withdraw temporarily from hemodialysis.

## Acknowledgments

We thank Mr. Kinichi Matsuyama and Ms. Mihoko Ishikawa in the Department of Pathology, Dokkyo Medical University, for their excellent technical help concerning immunohistochemistry, immunofluorescence and electron microscopy.

## Author contributions

**Conceptualization:** Akihiro Tojo.

**Data curation:** Mayu Uematsu-Uchida.

**Formal analysis:** Takehiro Ohira.

**Funding acquisition:** Toshihiko Ishimitsu.

**Investigation:** Shigeki Tomita, Akihiro Tojo.

**Methodology:** Akihiro Tojo.

**Project administration:** Akihiro Tojo.

**Resources:** Mayu Uematsu-Uchida, Takehiro Ohira.

**Software:** Hiroshi Satonaka.

**Supervision:** Toshihiko Ishimitsu.

**Validation:** Akihiro Tojo.

**Visualization:** Shigeki Tomita, Akihiro Tojo.

**Writing – original draft:** Mayu Uematsu-Uchida, Takehiro Ohira.

**Writing – review & editing:** Hiroshi Satonaka, Akihiro Tojo.
